# Chirality logic gates

**DOI:** 10.1126/sciadv.abq8246

**Published:** 2022-12-09

**Authors:** Yi Zhang, Yadong Wang, Yunyun Dai, Xueyin Bai, Xuerong Hu, Luojun Du, Hai Hu, Xiaoxia Yang, Diao Li, Qing Dai, Tawfique Hasan, Zhipei Sun

**Affiliations:** ^1^ Department of Electronics and Nanoengineering, Aalto University, Espoo 02150, Finland.; ^2^ QTF Centre of Excellence, Department of Applied Physics, Aalto University, Espoo 02150, Finland.; ^3^ Institute of Photonics and Photon Technology, Northwest University, Xi’an 710069, China.; ^4^ CAS Key Laboratory of Nanophotonic Materials and Devices, National Center for Nanoscience and Technology, Beijing 100190, China.; ^5^ Cambridge Graphene Centre, University of Cambridge, Cambridge CB3 0FA, UK.

## Abstract

The ever-growing demand for faster and more efficient data transfer and processing has brought optical computation strategies to the forefront of research in next-generation computing. Here, we report a universal computing approach with the chirality degree of freedom. By exploiting the crystal symmetry–enabled well-known chiral selection rules, we demonstrate the viability of the concept in bulk silica crystals and atomically thin semiconductors and create ultrafast (<100-fs) all-optical chirality logic gates (XNOR, NOR, AND, XOR, OR, and NAND) and a half adder. We also validate the unique advantages of chirality gates by realizing multiple gates with simultaneous operation in a single device and electrical control. Our first demonstrations of logic gates using chiral selection rules suggest that optical chirality could provide a powerful degree of freedom for future optical computing.

## Teaser

The photonic chirality logic gates show ultrafast, simultaneous multiple processing ability and electrical tunability.

## INTRODUCTION

Modern high-speed mobile networks and Internet of Things devices are generating exponentially increasing amounts of data. This has driven the rapid development of optical computing and processing because of their fully reconfigurable ability (*1*, *2*) and multifunction programmability (*3*). All-optical logic gates are indispensable components and have been demonstrated to perform all-optical calculations, including additions and multiplications (*4*). Current all-optical logic gates mainly use linear and nonlinear optical effects (*5*, *6*). With the linear optical approaches, common logic gates have been demonstrated in various optical structures, such as nanowire networks (*4*), photonic crystals (*7*, *8*), plasmonic waveguides (*9*–*12*), and diffractive metasurfaces (*13*). All-optical logic gates are also reported via nonlinear optical effects in different photonic structures, such as silicon resonators (*14*), waveguides (*15*), and photonic crystal structures (*16*, *17*).

Similar to different particles such as electrons and molecules, photons have an intrinsic degree of freedom, chirality (*17*). Optical chirality, defined by left-handed (σ^−^) and right-handed (σ^+^) circularly polarized light, has attracted huge interest in fundamental research and applications [e.g., quantum technology (*18*), chiral nonlinear optics (*19*), sensing (*20*), imaging (*21*), and valleytronics (*22*–*28*)]. Here, we report universal logic gates via nonlinear optics with the chirality degree of freedom. Exploiting the well-studied chiral selection rules enabled by threefold rotational symmetry in bulk silica and monolayer MoS_2_, we validate a range of ultrafast chirality logic gates (XNOR, NOR, AND, XOR, OR, and NAND) and a half adder. We also present the operation of multiple chirality logic gates simultaneously in a single device based on the broad bandwidth feature of phase matching–free nonlinear optics. Furthermore, we demonstrate electrical control of the chirality optical gates through valley exciton resonance in monolayer MoS_2_, offering exciting possibilities for electrically configurable optical computing.

## RESULTS

### Operation principle of the chirality logic gates

**Fig. 1.**
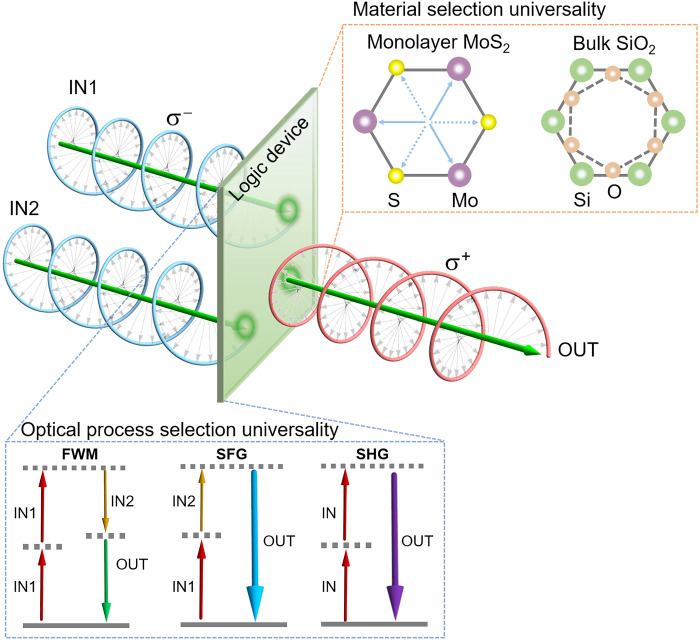
Illustration of the chirality logic gate and its concept universality in the selection of materials and optical processes. The generated output signal (i.e., OUT) in a nonlinear optical process is determined by the chirality of the two input beams (i.e., IN1 and IN2). The top right inset shows a top view of monolayer MoS_2_ and bulk SiO_2_ with threefold rotational symmetry to indicate the concept universality in the material selection. The bottom left inset presents the concept universality in the selection of nonlinear optical processes. IN and OUT denote the input and output beams, respectively.

Figure 1 shows the operation principle of logic gates with the chirality degree of freedom. Two optical beams (i.e., labeled as IN1 and IN2) with specific optical chirality (i.e., σ^−^ or σ^+^, considered as input logic 0/1) are incident onto a nonlinear optical material to implement the logic operation. According to the chirality of the two input beams, four input combinations, i.e., $\$(\sigma_{\mathrm{IN}1}^{-},\sigma_{\mathrm{IN}2}^{-})\$$, $\$(\sigma_{\mathrm{IN}1}^{-},\sigma_{\mathrm{IN}2}^{+})\$$, $\$(\sigma_{\mathrm{IN}1}^{+},\sigma_{\mathrm{IN}2}^{+})\$$, and $\$(\sigma_{\mathrm{IN}1}^{+},\sigma_{\mathrm{IN}2}^{-})\$$ [corresponding to (0,0), (0,1), (1,1), and (1,0)], are possible. The generated output signal (i.e., labeled as OUT in Fig. 1) is considered as logic 1/0 (based on the presence/absence of the output signal) in the nonlinear optical process (bottom left inset of Fig. 1).

Here, we present an example of using the chiral selection rules in nonlinear optics for the demonstration of the chirality logic gates. For example, nonlinear optics [e.g., four-wave mixing (FWM), sum-frequency generation (SFG), and second-harmonic generation (SHG)] in a material with threefold rotational symmetry under chiral incident light are governed by the angular momentum conservation (*29*)

$$\sigma_{\mathrm{OUT}}\hbar -\sigma_{\mathrm{IN}}\hbar =3N\hbar$$ (1)

where σ_OUT_ℏ and σ_IN_ℏ indicate the spin angular momentum of output and input beams (corresponding to the chirality), respectively; *N* is an integer; and 3*N*ℏ denotes the angular momenta of the crystal lattice originating from the threefold rotational symmetry. Therefore, the generated output signal in the nonlinear optical process (bottom left inset of Fig. 1) is determined by the chirality of the two input beams. Accordingly, chirality-based logic processing units can be constructed following the chiral selection rules (experimental demonstrations will be shown in the next section). We highlight that the chiral selection rule in the nonlinear optics (*26*–*29*) is enabled by the structure symmetry, fundamentally different from the valley-selective circular dichroism (e.g., photoluminescence) of transition metal dichalcogenides (*22*–*25*).

### Experimental demonstration of universal chirality logic gates

**Fig. 2.**
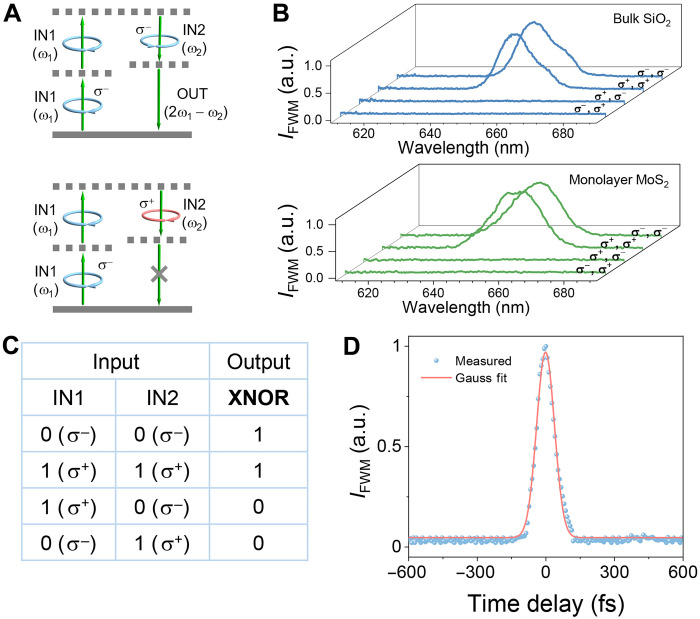
Chirality XNOR logic gate. (**A**) The optical chirality–dependent selection rule in FWM due to threefold rotational symmetry in materials. (**B**) The normalized FWM output spectra in bulk SiO_2_ and monolayer MoS_2_ under the excitation of two input beams with different chirality combinations. (**C**) The truth table of the chirality XNOR logic gate. (**D**) The normalized cross-correlation spectrum of the FWM signal in MoS_2_ with a Gauss fit. a.u., arbitrary units.

As an example to demonstrate the chirality logic gate operation principle (Fig. 1), we first demonstrate an XNOR logic gate as discussed above with FWM in bulk silica and monolayer MoS_2_, because of their chiral selection rules of the nonlinear optics enabled by the threefold rotational symmetry in their crystal lattice (top right inset of Fig. 1) (*30*). However, we note that the general concept and demonstration here can be applied not only to other transition metal dichalcogenides and their heterostructures (*31*) but also to other materials and even structures [e.g., hybrid metamaterials (*32*) and metasurfaces (*33*)] with threefold rotational symmetry. Details on the possibility of applying the operation principle to materials with different rotational symmetries are given in table S1. Figure 2A illustrates the energy level diagram of a degenerate FWM process and the chiral selection rules in bulk silica and monolayer MoS_2_, where two input beams (i.e., IN1 and IN2) at the frequency of ω_1_ and ω_2_ produce the output beam (i.e., OUT) at the frequency of 2ω_1_ − ω_2_. Figure 2B shows the measured FWM spectra under different input chirality conditions when the wavelengths of the two input beams are ~800 nm (~1.5 μW) and 1036 nm (~8.5 μW). Note that all output spectra are measured under incident beams with four different chirality combinations in a real case (experimental setup and sample fabrication are given in Materials and Methods). According to the chiral selection rules (Fig. 2A) enabled by threefold rotational symmetry in bulk silica and monolayer MoS_2_, the generated FWM peak appears (logic 1) at ~651 nm, only when both the two input beams have the same chirality (e.g., σ^−^ or σ^+^). Here, the conversion efficiency of FWM is ~3.4 × 10^−7^% and third-order susceptibility $\$|\chi_{\mathrm{eff}}^{\left(3\right)}|\$$ is ~1.9 × 10^−19^ m^2^/V^2^. When the two input circularly polarized beams have opposite chirality (i.e., one with σ^+^ and the other with σ^−^), FWM is not observed (logic 0). Therefore, as discussed above, if the chirality of the input beams is defined as a value of 0 and 1 (corresponding to σ^−^ and σ^+^ circular polarizations) and the presence or absence of the FWM signal at the output is considered as logic 1 or 0, respectively, an XNOR logic gate is realized. The results in bulk SiO_2_ (Fig. 2B, top) and monolayer MoS_2_ (Fig. 2B, bottom) all agree well with the theoretical chiral selection rule in Fig. 2A and confirm the XNOR logic gate operation. The truth table of the demonstrated chirality XNOR gate with corresponding inputs and outputs is shown in Fig. 2C. Note that the gate operation in bulk SiO_2_ and monolayer MoS_2_ underlines the universality of the chirality gate concept in the material selection.

Figure 2D shows the cross-correlation FWM spectrum of the two input beams (the pulse width of the two input beams is ~60 fs) in MoS_2_, indicating the operation speed information of the logic gate. The full width at half maximum of the cross-correlation FWM spectra is ~78 fs, comparable with the input pulse width. This reflects the intrinsic ~femtosecond ultrafast response (*34*) of the logic gate far beyond our measurement resolution limited by the input pulse width. Such an ultrafast operation speed, combined with two-dimensional (2D) atomic thickness, makes our approach ideal for future ultrafast nanophotonic on-chip applications.

We note that the same chirality logic gate concept can be applied to other nonlinear optical processes beyond FWM. For example, we experimentally construct an XNOR gate with SFG (see fig. S3) with the same incident wavelengths as FWM and Buffer and NOT gates with SHG with an incident beam at 800 nm (~1.5 μW) in monolayer MoS_2_ (see fig. S5). This also underlines the universal applicability of the chirality logic gate concept with different nonlinear optical processes.

### Diversity of chirality logic gates

**Fig. 3.**
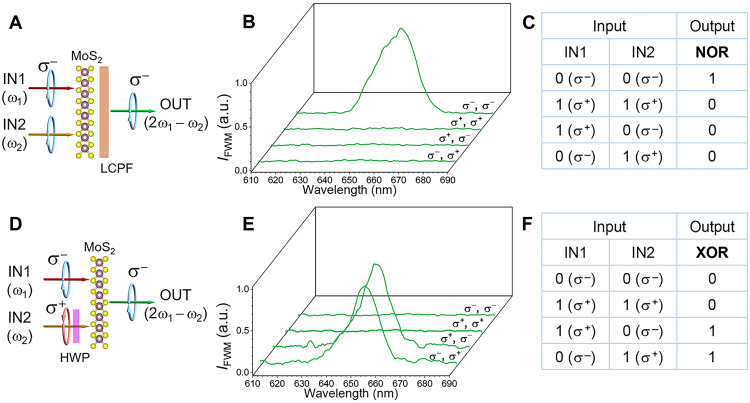
Diversity of chirality logic gates. (**A**) Schematic illustration of NOR gate. (**B**) Output FWM spectra behind LCPF. (**C**) Truth table of NOR chirality logic gate. (**D**) Schematic illustration of XOR gate. (**E**) Output FWM spectra after the insertion of the half–wave plate in input beam 2. (**F**) Truth table of XOR chirality logic gate. The similar concept applies to bulk SiO_2_.

Using the same basic device structure in the example of monolayer MoS_2_, we also realize different chirality logic gates by taking advantage of the fact that the optical chirality can be flexibly changed with linear optical components. As shown in fig. S6A, the generated FWM presents different chirality based on the chiral selection rules. When the two input beams are of the same σ^−^ (σ^+^) circular polarization, the generated FWM has the same σ^−^ (σ^+^) chirality. Therefore, a chirality NOR logic gate can be realized (Fig. 3A) after inserting a left-handed circular polarization filter (LCPF), consisting of a quarter–wave plate and a polarizer. The generated FWM signal with σ^−^ chirality when the input beams have σ^−^ circular polarization can go through the LCPF (logic 1). On the other hand, when the input beams have σ^+^ circular polarization, the generated FWM signal with σ^+^ chirality will be blocked by the LCPF, giving a logic 0 output. Figure 3B shows the measured FWM spectra behind LCPF (Fig. 3A) with different input conditions. In this case, the system (i.e., MoS_2_ + LCPF; Fig. 3A) represents a NOR logic gate (truth table shown in Fig. 3C). Note that by inserting a right-handed circular polarization filter (RCPF) behind MoS_2_, only FWM with σ^+^ chirality can be observed when the two input beams are of the same σ^+^ circular polarization. Thus, a chirality AND logic gate can be readily constructed using the same operation principle.

Exploiting a similar concept, we also construct a chirality XOR logic gate based on FWM. In this instance, we insert a half–wave plate in the path of input beam 2 to flip its input chirality (Fig. 3D) before the monolayer MoS_2_. Figure 3E shows the measured FWM spectra after the insertion of the half–wave plate. Because of the flipped chirality of input beam 2, we can only observe the FWM spectra when the two inputs have opposite chirality, different from the previous XNOR gate case (Fig. 2B). When one input beam is σ^−^ (σ^+^) circularly polarized and the other input beam is σ^+^ (σ^−^) circularly polarized, the generated FWM appears with σ^−^ (σ^+^) chirality (see fig. S6B). Therefore, a chirality XOR logic gate is constructed (truth table shown in Fig. 3F). On the basis of the same working principle, we also present the designs of chirality OR and NAND logic gates and a half adder in figs. S8 and S9. The demonstrations and proposed designs offer the possibility of constructing complex logic expressions and networks with our chirality concept. These logic gates could also be readily constructed using bulk SiO_2_.

### Simultaneous multiple chirality logic gates

**Fig. 4. F4:**
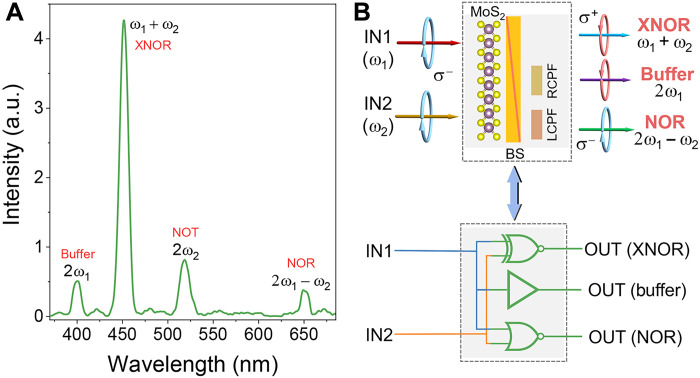
Simultaneous construction of multiple chirality logic gates. (**A**) Simultaneous observation of SHG, SFG, and FWM spectra and their corresponding chirality logic gates. The two input beams are ω_1_ (~800 nm) and ω_2_ (~1036 nm) with σ^−^ polarization. (**B**) The simplified schematic illustration of the logic gates by inserting a beam splitter (BS) and different filters after monolayer MoS_2_ (top) and its equivalent logic gate network (bottom).

We next demonstrate the simultaneous construction and operation of multiple chirality logic gates in parallel by using the broadband feature of phase matching–free nonlinear optics in the MoS_2_ monolayer. Figure 4A shows the measured SHG, SFG, and FWM spectra from monolayer MoS_2_ when the input beams have both σ^−^ circular polarization with ω_1_ (~800 nm, ~1.5 μW) and ω_2_ (~1036 nm, ~8.5 μW). There are four peaks, including two SHG peaks at 2ω_1_ (~400 nm) and 2ω_2_ (~518 nm), an SFG peak at ω_1_ + ω_2_ (~451 nm), and an FWM peak at 2ω_1_ − ω_2_ (~651 nm), respectively. The SFG peak is stronger than the SHG peaks, mainly because both input beams contribute to the SFG process and result in higher input power. The FWM peak is lower since it is a third-order nonlinear process, which is typically smaller than the second-order nonlinear process (e.g., SHG and SFG). As discussed, SHG, SFG, and FWM in monolayer MoS_2_ can be used to construct various chirality logic gates (Figs. 2 and 3 and figs. S3 to S5). Therefore, we present a schematic design (Fig. 4B) to simultaneously realize various chirality logic gates in parallel by inserting a beam splitter behind the MoS_2_ monolayer to detect the SFG, FWM, and SHG signals simultaneously, i.e., the multiple chirality XNOR, NOR, and Buffer (NOT) logic gates (labeled in Fig. 4A). Note that another beam splitter[Fig F4], an LCPF, and an RCPF are also added behind the beam splitter to realize the Buffer (NOT) gate (as shown in fig. S5D) and NOR gate (as shown in Fig. 3A) simultaneously. Such simultaneous construction of multiple logic gates in a single device is fundamentally different from the previously demonstrated parallel electronic and optical logic gates, which typically show only one logic operation capability.

We also demonstrate simultaneous multiple chirality logic gates at other input wavelengths (results with ω_1_ ~ 800 nm and ω_2_ ~ 860 nm in fig. S7). This can prove the wavelength-independent nature of our concept, which verifies that the well-established chiral selection rule of the nonlinear process (e.g., FWM, SFG, and SHG) is based on angular momentum conservation enabled by the crystal symmetry (*27*–*29*). Thus, our proposed chirality logic gate, in principle, can work without bandwidth limitation, which is beneficial for logic circuits. Notably, this is unlike the previously demonstrated nonlinear optical logic gates and the recently proposed valley-selective linear optical logic gates (*25*) that typically work at specific wavelengths. We further note that similar results [e.g., simultaneous observation of SHG, SFG, and FWM (*35*); simultaneous observation of SHG and difference frequency generation (*36*); and simultaneous observation of SHG, third-harmonic generation, and higher-order harmonic generation (**37**)] have been reported before with linearly polarized pump beams. Therefore, in principle, our simultaneous construction of multiple logic gates with different nonlinear optical processes at a broad input wavelength range fully opens up the possibility of fabricating complex chirality logic gate networks. We additionally note that all the logic device concepts demonstrated above can be realized using bulk/thin-film materials with threefold rotational symmetry, making this approach to optical computing universal and not confined to a single active material.

### Electrical control of the chirality logic gates

Traditionally, it is challenging to control optical gates electronically. Recently, it has been reported that electrically tuned SHG (**38**) and FWM (*35*) in monolayer transition metal dichalcogenides on exciton resonance with linearly polarized pump beams. Here, we extend the same mechanism from the linearly polarized light (**35**,**38**) to circularly polarized light and demonstrate an electrically reconfigurable chirality logic gate. This offers a practical approach to configure the chirality logic gate concept demonstrated here with the valley degree of freedom for interfacing between electrical and optical computing.

**Fig. 5.**
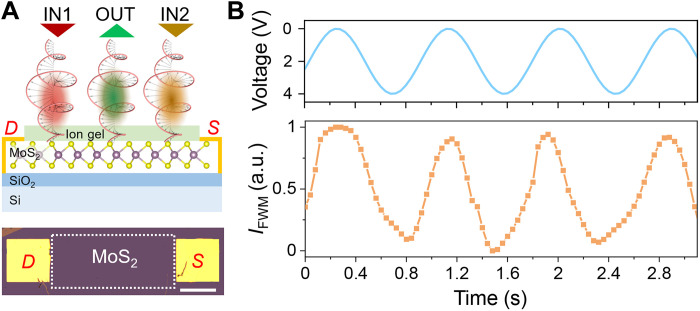
Proof-of-concept demonstration of electrically controllable chirality logic gate. (**A**) Schematic of the chirality logic gate based on a MoS_2_ field-effect transistor with two σ^+^ circularly polarized incident beams (top) and one σ^+^ circularly polarized output beam, where *S*, *D*, and *G* denote the source, drain, and gate electrodes, respectively. The ion gel acts as the gate dielectric between the gate and source electrodes. Bottom denotes the optical image of the monolayer MoS_2_ channel (white rectangle) with source-drain contacts on the SiO_2_ substrate. Scale bar, 250 μm. (**B**) The modulated peak intensity of FWM (orange curve) under the gate voltage (blue curve). The two input beams are ~800 and 1036 nm with σ^+^ polarization. The results are rescaled through min-max normalization.

Figure 5A shows the schematic illustration of the proof-of-concept demonstration of an electrically tunable chirality logic gate with monolayer MoS_2_ and the optical image of the monolayer MoS_2_ channel with drain (*D*) and source (*S*) electrodes. Detailed fabrication of the gated MoS_2_ device is given in Materials and Methods. We carry out the chirality logic gate experiment when the input beams both have σ^+^ circular polarization at the frequencies of ω_1_ (~800 nm, ~1.5 μW) and ω_2_ (~1036 nm, ~8.5 μW), which can generate an FWM peak at 2ω_1 _− ω_2_ (~651 nm) around A-exciton of monolayer MoS_2_ (see fig. S10). Figure 5B shows the measured peak intensity of the FWM signal (bottom) under the gate voltage modulation (top). As observed, the FWM signal changes with the external voltage, which is caused by the reduced exciton oscillation strength with the extra injected electrons (*35*, *38*). The nonuniform modulation depth of the FWM signal is likely caused by the degradation and the heat effect of the gate material ion gel under the external gate voltage, which also limits the response of the demonstration. In principle, the demonstrated fast (up to a few tens of gigahertz) MoS_2_ transistors can possibly enable high-speed electrical control. Nevertheless, the electrical modulation of chirality logic gates sheds light on the interfacing between electrical and optical computing toward next-generation mixed and hybrid computing.

The ON/OFF ratio of a logic gate is of paramount importance for building logic networks. In our chirality logic gates, the chirality of the output signal, as an excellent alternative to conventional electrically controlled logic gates, is determined by the inherent material characteristics, such as inversion symmetry breaking (*39*) or threefold rotational symmetry of monolayer MoS_2_ (*30*). As demonstrated, the output signal intensity is absent when the two inputs have opposite chirality. This indicates that the output ON/OFF states of the chirality logic gate could be unambiguously identified for applications.

### Future perspectives

The speed advantage of optical computing is a substantial motivation for developing all-optical logic gates. In our measurement, the speed of our chirality logic gate is less than ~100 fs, which is mainly limited by the input pulse duration (Fig. 2D). Note that the nonlinear optical effects (such as FWM and SFG) are coherent and typically occur at ~tens of femtoseconds (*34*). This indicates that such coherent optical processes allow much faster operation speed.

Recently, optical chirality has been successfully used to address valley polarization and chiral photoluminescence in monolayer semiconductors and their heterostructures because of symmetry breaking (*22*–*24*). In principle, such unique valley-based linear optical properties can also enable multifunctional logic gates via noncoherent photoluminescence and absorption effects (*25*). In contrast, our demonstrated chirality logic gates with nonlinear optics offer advantages of atomically thin footprint, multiple processing, electrical tunability with valley, and ultrafast processing speed across a wide range of wavelengths. Furthermore, the logic gate concept can be enriched with various operational ideas in 2D materials and their heterostructures [e.g., the interfacing between electronic and optical gates via spin-orbit coupling (*40*) or spin-valley diffusion (*41*) and optical memories with long-lived valley lifetime (*42*)].

Toward future practical applications, we need to enhance the nonlinear conversion efficiency. We note that high nonlinear optical conversion efficiency has been reported in 2D materials [e.g., up to >0.2% in NbOI_2_ nanosheets (*43*)], which shows possibilities of further increasing the conversion efficiency with suitable 2D materials for practical applications. As demonstrated here, the operation principle also applies to bulk materials, which enriches the highly nonlinear optical material selection option. Furthermore, in principle, we can use various enhancement strategies, such as cavities (*44*), microstructures [e.g., photonic crystal structures (*45*) and metastructures (*46*)], and energy transfer method (*47*), to boost the nonlinear optical conversion efficiency.

As mentioned, the operation principle is governed by the angular momentum conservation law determined by the symmetry of the crystal structure (*27*–*29*). This fully indicates the wavelength-independent nature of our concept, which is beneficial for future successive operations. Besides, the demonstrated simultaneous operation of multiple chirality logic gates in a single device, in notable contrast to the conventional optical and electrical logic devices that typically perform one logic operation per device, can possibly enable an easy way to perform multifunctional logic circuits and networks. Furthermore, we also believe that various integrated platforms [e.g., optical fibers (*45*) and silicon waveguides (*15*)] will not only simplify the current free-space design used in our proof-of-principle demonstration but also enhance the nonlinear optical conversion efficiency for future large-scale integrated applications.

## DISCUSSION

In conclusion, we reported a universal computing approach with the chirality degree of freedom. Different ultrafast chirality logic gates (XNOR, NOR, AND, XOR, OR, and NAND) and a half adder have been reported on the basis of the optical chirality–dependent selection rules. Because of the broadband feature of the phase matching–free nonlinear optics in the atomically thin layer, multifunction logic gates capable of simultaneous logic operation have also been realized in a single device. Our chirality logic gate mechanism with a versatile selection of active materials, ultrafast processing speed, and electrical tunability may be extended to many data-heavy applications, such as on-chip integrated signal and video processing across a wide spectral range.

## MATERIALS AND METHODS

### Chiral selection rule

According to the conservation of angular momentum in nonlinear optics such as third-harmonic generation (*27*), SFG (*28*), SHG (*43*), and two-photon photoluminescence processes (*26*), the allowed nonlinear processes by threefold rotational symmetry can be expressed as σ_OUT_ℏ − σ_IN_ℏ = 3*N*ℏ. As shown in the top inset of Fig. 1A, the crystal lattice of monolayer MoS_2_ and bulk SiO_2_ belongs to the threefold rotational symmetry. Therefore, (i) for the FWM, the angular momentum conservation of a four-photon process can be written as σ_(2ω_1_ − ω_2_)_ℏ − 2σ_ω_1__ℏ + σ_ω_2__ℏ = 3*N*ℏ. As a result, the chiral selection rule indicates that when the two input beams have the same chirality, the FWM beam with the same chirality will be generated. FWM is forbidden when the two input beams have opposite chirality; (ii) for the SFG, the angular momentum conservation of a three-photon process can be written as σ_(ω_1_ + ω_2_)_ℏ − σ_ω_1__ℏ − σ_ω_2__ℏ = 3*N*ℏ. The chiral selection rule leads to the SFG beam with the opposite chirality allowed to emit when the two input beams have the same chirality, and SFG is forbidden when the two input beams have opposite chirality; (iii) for the SHG, the angular momentum conservation of a three-photon process can be written as σ_2ω_1__ℏ − 2σ_ω_1__ℏ = 3*N*ℏ. The chiral selection rule implies that the SHG beam always has opposite chirality to the input beam.

### SHG, SFG, and FWM measurements

A laser source with a repetition rate of 2 kHz from an amplified Ti:sapphire femtosecond laser system (Spectra-Physics Solstice Ace) generates the two input beams for the experiments (see fig. S1). The polarization orientation of input beam 1 is controlled by rotating the half–wave plate. After passing through a delay line system, input beam 2 is collinearly combined with input beam 1 by a dichroic mirror in the time and spatial domains. Subsequently, the two linearly polarized input beams are converted into circularly polarized beams by the quarter–wave plate and then focused onto the MoS_2_ monolayer by a 40× objective lens [numerical aperture (NA) = 0.75, Nikon]. The generated signal pulses, including SHG, SFG, and FWM, are collected by another 40× objective lens (NA = 0.5, Nikon), which leads to a spectrometer (Andor) equipped with a photomultiplier tube (Hamamatsu) connected to a lock-in amplifier (Stanford Research Systems) to detect the generated signals. Two 700-nm short-pass filters (Edmund) after the output objective lens are used to cut off the input pulses. A combination of a quarter–wave plate and a polarizer is used to analyze the chirality of the signal pulses before the detector.

### Electrically modulated FWM from MoS_2_ device

For the measurement of the FWM signal under an external voltage, the transmission configuration in fig. S1 is changed into a reflective configuration (*35*), where a beam splitter is inserted between a dichroic mirror and the first objective lens (Obj. 1) to deflect the signal beam from the MoS_2_ device into a spectrometer. Figure S10 shows the measured FWM spectra under an external voltage *V*_g_ range of 4 or −4 V.

### Sample preparation

Monolayer MoS_2_ flakes are grown on a quartz substrate with a thickness of 500 μm by chemical vapor deposition (CVD). A Na_2_MoO_4_ aqueous solution (5 mg/ml) is spin-coated on the surface of the substrate. The substrate is then heated at 800°C, and ~10 mg of sulfur is heated at 170°C for 5 min in a high-purity argon atmosphere. The optical microscope image, Raman, photoluminescence, and absorption spectra are provided in fig. S2 to identify high-quality monolayer MoS_2_ flakes. The quartz used for the logic gate is *c*-axis cut (Siegert wafer).

### Characterization of monolayer MoS_2_

Figure S2A gives the optical image of the monolayer MoS_2_ flake on the quartz substrate, which shows a typical triangular shape. Figure S2B shows the Raman spectrum of the monolayer MoS_2_ measured with a continuous-wave laser at 532 nm. The Raman spectrum shows two characteristic peaks, which represent the in-plane mode (E_2g_) and out-of-plane mode (A_1g_) at ∼384.4 and ∼404.4 cm^−1^. Figure S2C shows the photoluminescence spectrum of monolayer MoS_2_ measured with the laser at 532 nm. The photoluminescence spectrum exhibits two characteristic peaks corresponding to the A-exciton (at ~679.9 nm) and B-exciton (at ~628.7 nm), respectively. Figure S2D shows the linear absorption spectrum in the visible range, which presents three characteristic peaks denoting the A-exciton (at ~678.6 nm), B-exciton (at ~629.7 nm), and C-exciton (at ~433.5 nm), respectively. The measured results demonstrate the good quality of our monolayer MoS_2_.

### MoS_2_ transistor fabrication

Monolayer MoS_2_ grown on a SiO_2_/Si substrate by the CVD method is used for the gated device. We pattern the source, drain, and gate electrodes using electron-beam lithography (Vistec) and then evaporate Ti/Au (5/50 nm) using an electron-beam evaporator. A drop of the ion-gel solution is cast onto the MoS_2_ flake and the electrodes. The ion-gel solution is a mixture of 22 mg polystyrene-*b*-poly(ethylene oxide)-*b*-polystyrene (Polymer Source), 0.56 g 1-ethyl-3-methylimidazolium bis(trifluoromethylsulfonyl)imide (Sigma-Aldrich), and 20 ml anhydrous dichloromethane (Sigma-Aldrich). All electrodes are wire-bonded to a printed circuit board for external electrical control by source meters.

## Notes

View/request a protocol for this paper from *Bio-protocol*.

## References

[1] W. Liu, M. Li, R. S. Guzzon, E. J. Norberg, J. S. Parker, M. Lu, L. A. Coldren, J. Yao,A fully reconfigurable photonic integrated signal processor. Nat. Photon. 10,190–195 (2016).

[2] E. N. Mohammadi, B. Edwards, N. Engheta,Inverse-designed metastructures that solve equations. Science 363,1333–1338 (2019). 30898930 10.1126/science.aaw2498

[3] D. Perez-Lopez, A. Lopez, P. DasMahapatra, J. Capmany,Multipurpose self-configuration of programmable photonic circuits. Nat. Commun. 11,6359 (2020). 33311499 10.1038/s41467-020-19608-w PMC7733469

[4] H. Yang, V. Khayrudinov, V. Dhaka, H. Jiang, A. Autere, H. Lipsanen, Z. Sun, H. Jussila,Nanowire network–based multifunctional all-optical logic gates. Sci. Adv. 4,eaar7954 (2018). 30062123 10.1126/sciadv.aar7954 PMC6063535

[5] P. Singh, D. K. Tripathi, S. Jaiswal, H. K. Dixit,All-optical logic gates: Designs, classification, and comparison. Adv. Opt. Technol. 2014,275083 (2014).

[6] Y. Chen, Y. Cheng, R. Zhu, F. Wang, H. Cheng, Z. Liu, C. Fan, Y. Xue, Z. Yu, J. Zhu, X. Hu, Q. Gong,Nanoscale all-optical logic devices. Sci. China Phys. Mech. Astron. 62,044201 (2019).

[7] Y. Zhang, Y. Zhang, B. Li,Optical switches and logic gates based on self-collimated beams in two-dimensional photonic crystals. Opt. Express 15,9287–9292 (2007). 19547271 10.1364/oe.15.009287

[8] Y. Fu, X. Hu, Q. Gong,Silicon photonic crystal all-optical logic gates. Phys. Lett. A 377,329–333 (2013).

[9] H. Wei, Z. Wang, X. Tian, M. Käll, H. Xu,Cascaded logic gates in nanophotonic plasmon networks. Nat. Commun. 2,387 (2011). 21750541 10.1038/ncomms1388 PMC3144585

[10] H. Wei, Z. Li, X. Tian, Z. Wang, F. Cong, N. Liu, S. Zhang, P. Nordlander, N. J. Halas, H. Xu,Quantum dot-based local field imaging reveals plasmon-based interferometric logic in silver nanowire networks. Nano Lett. 11,471–475 (2011). 21182282 10.1021/nl103228b

[11] B. Piccione, C. H. Cho, L. K. Van Vugt, R. Agarwal,All-optical active switching in individual semiconductor nanowires. Nat. Nanotechnol. 7,640–645 (2012). 22941404 10.1038/nnano.2012.144

[12] C. Peng, J. Li, H. Liao, Z. Li, C. Sun, J. Chen, Q. Gong,Universal linear-optical logic gate with maximal intensity contrast ratios. ACS Photon. 5,1137–1143 (2018).

[13] C. Qian, X. Lin, X. Lin, J. Xu, Y. Sun, E. Li, B. Zhang, H. Chen,Performing optical logic operations by a diffractive neural network. Light Sci. Appl. 9,59 (2020). 32337023 10.1038/s41377-020-0303-2 PMC7154031

[14] Q. Xu, M. Lipson,All-optical logic based on silicon micro-ring resonators. Opt. Express 15,924–929 (2007). 19532318 10.1364/oe.15.000924

[15] S. Gao, X. Wang, Y. Xie, P. Hu, Q. Yan,Reconfigurable dual-channel all-optical logic gate in a silicon waveguide using polarization encoding. Opt. Lett. 40,1448–1451 (2015). 25831356 10.1364/OL.40.001448

[16] Z. Zhu, W. Ye, J. Ji, X. Yuan, C. Zen,High-contrast light-by-light switching and AND gate based on nonlinear photonic crystals. Opt. Express 14,1783–1788 (2006). 19503506 10.1364/oe.14.001783

[17] J. Mun, M. Kim, Y. Yang, T. Badloe, J. Ni, Y. Chen, C. Qiu, J. Rho,Electromagnetic chirality: From fundamentals to nontraditional chiroptical phenomena. Light Sci. Appl. 9,139 (2020). 32922765 10.1038/s41377-020-00367-8 PMC7463035

[18] P. Lodah, S. Mahmoodian, S. Stobbe, A. Rauschenbeute, P. Schneeweiss, J. Volz, H. Pichler, P. Zoller,Chiral quantum optics. Nature 541,473–480 (2017). 28128249 10.1038/nature21037

[19] Y. Zhang, X. Bai, J. Arias Muñoz, Y. Dai, S. Das, Y. Wang, Z. Sun,Coherent modulation of chiral nonlinear optics with crystal symmetry. Light Sci. Appl. 11,216 (2022). 35803908 10.1038/s41377-022-00915-4 PMC9270472

[20] S. Yoo, Q. H. Park,Metamaterials and chiral sensing: A review of fundamentals and applications. Nanophotonics 8,249–261 (2019).

[21] M. Khorasaninejad, W. Chen, A. Zhu, J. Oh, R. C. Devlin, D. Rousso, F. Capasso,Multispectral chiral imaging with a metalens. Nano Lett. 16,4595–4600 (2016). 27267137 10.1021/acs.nanolett.6b01897

[22] T. Cao, G. Wang, W. Han, H. Ye, C. Zhu, J. Shi, Q. Niu, P. Tan, E. Wang, B. Liu, J. Feng,Valley-selective circular dichroism of monolayer molybdenum disulphide. Nat. Commun. 3,887 (2012). 22673914 10.1038/ncomms1882 PMC3621397

[23] K. F. Mak, K. He, J. Shan, T. F. Heinz,Control of valley polarization in monolayer MoS_2_ by optical helicity. Nat. Nanotechnol. 7,494–498 (2012). 22706698 10.1038/nnano.2012.96

[24] H. Zeng, J. Dai, W. Yao, D. Xiao, X. Cui,Valley polarization in MoS_2_ monolayers by optical pumping. Nat. Nanotechnol. 7,490–493 (2012). 22706701 10.1038/nnano.2012.95

[25] Y. Wang, C. Luo, A. Yabushita, K. Wu, T. Kobayashi, C. Chen, L. Li,Ultrafast multi-level logic gates with spin-valley coupled polarization anisotropy in monolayer MoS_2_. Sci. Rep. 5,8289 (2015). 25656222 10.1038/srep08289 PMC4319162

[26] J. Xiao, Z. Ye, Y. Wang, H. Zhu, Y. Wang, X. Zhang,Nonlinear optical selection rule based on valley-exciton locking in monolayer WS_2_. Light Sci. Appl. 4,e366 (2015).

[27] J. Cheng, D. Huang, T. Jiang, Y. Shan, Y. Li, S. Wu, W. Liu,Chiral selection rules for multi-photon processes in two-dimensional honeycomb materials. Opt. Lett. 44,2141–2144 (2019). 31042168 10.1364/OL.44.002141

[28] K. Yao, E. Yanev, H. Chuang, M. R. Rosenberger, X. Xu, T. Darlington, K. M. McCreary, A. T. Hanbicki, K. Watanabe, T. Taniguchi, B. T. Jonker, X. Zhu, D. N. Basov, J. C. Hone, P. J. Schuck,Continuous wave sum frequency generation and imaging of monolayer and heterobilayer two-dimensional semiconductors. ACS Nano 14,708–714 (2020). 31891477 10.1021/acsnano.9b07555

[29] C. L. Tang, H. Rabin,Selection rules for circularly polarized waves in nonlinear optics. Phys. Rev. B 3,4025–4034 (1971).

[30] T. Y. Li, Y. Rao, K. F. Mak, Y. You, S. Wang, C. R. Dean, T. F. Heinz,Probing symmetry properties of few-layer MoS_2_ and h-BN by optical second-harmonic generation. Nano Lett. 13,3329–3333 (2013). 23718906 10.1021/nl401561r

[31] T. Chowdhury, E. C. Sadler, T. J. Kempa,Progress and prospects in transition-metal dichalcogenide research beyond 2D. Chem. Rev. 120,12563–12591 (2020). 32960576 10.1021/acs.chemrev.0c00505

[32] U. Kilic, M. Hilfiker, A. Ruder, R. Feder, E. Schubert, M. Schubert, C. Argyropoulos,Broadband enhanced chirality with tunable response in hybrid plasmonic helical metamaterials. Adv. Funct. Mater. 31,2010329 (2021).

[33] M. Manjappa, P. Pitchappa, N. Singh, N. Wang, N. I. Zheludev, C. Lee, R. Singh,Reconfigurable MEMS Fano metasurfaces with multiple-input-output states for logic operations at terahertz frequencies. Nat. Commun. 9,4056 (2018). 30283070 10.1038/s41467-018-06360-5 PMC6170453

[34] R. W. Boyd, *Nonlinear Optics* (Academic Press, 2020).

[35] Y. Dai, Y. Wang, S. Das, H. Xue, X. Bai, E. Hulkko, G. Zhang, X. Yang, Q. Dai, Z. Sun,Electrical control of interband resonant nonlinear optics in monolayer MoS_2_. ACS Nano 14,8442–8448 (2020). 32598130 10.1021/acsnano.0c02642 PMC7735744

[36] Y. Wang, M. Ghotbi, S. Das, Y. Dai, S. Li, X. Hu, X. Gan, J. Zhao, Z. Sun,Difference frequency generation in monolayer MoS_2_. Nanoscale 12,19638–19643 (2020). 32524108 10.1039/d0nr01994a

[37] A. Säynätjoki, L. Karvonen, H. Rostami, A. Autere, S. Mehravar, A. Lombardo, R. A. Norwood, T. Hasan, N. Peyghambarian, H. Lipsanen, K. Kieu, A. C. Ferrari, M. Polini, Z. Sun,Ultra-strong nonlinear optical processes and trigonal warping in MoS_2_ layers. Nat. Commun. 8,893 (2017). 29026087 10.1038/s41467-017-00749-4 PMC5715017

[38] K. L. Seyler, J. R. Schaibley, P. Gong, P. Rivera, A. M. Jones, S. Wu, J. Yan, D. G. Mandrus, W. Yao, X. Xu,Electrical control of second-harmonic generation in a WSe_2_ monolayer transistor. Nat. Nanotechnol. 10,407–411 (2015). 25895004 10.1038/nnano.2015.73

[39] L. Du, T. Hasan, A. Castellanos-Gomez, G. Liu, Y. Yao, C. N. Lau, Z. Sun,Engineering symmetry breaking in 2D layered materials. Nat. Rev. Phys. 3,193–206 (2021).

[40] D. Unuchek, A. Ciarrocchi, A. Avsar, K. Watanabe, T. Taniguchi, A. Kis,Room-temperature electrical control of exciton flux in a van der Waals heterostructure. Nature 560,340–344 (2018). 30046107 10.1038/s41586-018-0357-y

[41] M. Onga, Y. Zhang, T. Ideue, Y. Iwasa,Exciton Hall effect in monolayer MoS_2_. Nat. Mater. 16,1193–1197 (2017). 28967914 10.1038/nmat4996

[42] C. Jiang, W. Xu, A. Rasmita, Z. Huang, K. Li, Q. Xiong, W. Gao,Microsecond dark-exciton valley polarization memory in two-dimensional heterostructures. Nat. Commun. 9,753 (2018). 29467477 10.1038/s41467-018-03174-3 PMC5821860

[43] I. Abdelwahab, B. Tilmann, Y. Wu, D. Giovanni, I. Verzhbitskiy, M. Zhu, R. Berté, F. Xuan, L. S. Menezes, G. Eda, T. Sum, S. Quek, S. A. Maier, K. P. Loh,Giant second-harmonic generation in ferroelectric NbOI_2_. Nat. Photon. 16,644–650 (2022).

[44] X. Liu, T. Galfsky, Z. Sun, F. Xia, E. Lin, Y. Lee, S. Kéna-Cohen, V. M. Menon,Strong light–matter coupling in two-dimensional atomic crystals. Nat. Photon. 9,30–34 (2015).

[45] Y. Zuo, W. Yu, C. Liu, X. Cheng, R. Qiao, J. Liang, X. Zhou, J. Wang, M. Wu, Y. Zhao, P. Gao, S. Wu, Z. Sun, K. Liu, X. Bai, Z. Liu,Optical fibres with embedded two-dimensional materials for ultrahigh nonlinearity. Nat. Nanotechnol. 15,987–991 (2020). 32958935 10.1038/s41565-020-0770-x

[46] Y. Dai, Y. Wang, S. Das, S. Li, H. Xue, A. Mohsen, Z. Sun,Broadband plasmon-enhanced four-wave mixing in monolayer MoS_2_. Nano Lett. 21,6321–6327 (2021). 34279968 10.1021/acs.nanolett.1c02381 PMC8323120

[47] H. Hong, C. Wu, Z. Zhao, Y. Zuo, J. Wang, C. Liu, J. Zhang, F. Wang, J. Feng, H. Shen, J. Yin, Y. Wu, Y. Zhao, K. Liu, P. Gao, S. Meng, S. Wu, Z. Sun, K. Liu, J. Xiong,Giant enhancement of optical nonlinearity in two-dimensional materials by multiphoton-excitation resonance energy transfer from quantum dots. Nat. Photon. 15,510–515 (2021).

