# Supplementary material for: Chirality logic gates
Source: Sci Adv. 2022 Dec 9;8(49):eabq8246. doi: 10.1126/sciadv.abq8246 (PMC9733934; doi:10.1126/sciadv.abq8246)
Supplement: Supplementary file 1 — Table S1 Figs. S1 to S11 [file sciadv.abq8246_sm.pdf]

Science Advances
AAAS

Supplementary Materials for

Chirality logic gates

Yi Zhang *et al.*

Corresponding author: Zhipei Sun, zhipei.sun@aalto.fi

Sci. Adv. **8**, eabq8246 (2022)
DOI: 10.1126/sciadv.abq8246

This PDF file includes:

Table S1
Figs. S1 to S11

1. Angular momentum conservation in crystals with different rotational symmetries

Table S1 Generation of typical nonlinear optical processes with different circularly polarized incidents in crystals with different rotational symmetries

Rotational symmetry	Incident combinations	FWM	SFG	SHG
Two-fold	$(\sigma_{\mathrm{IN1}}^{-}, \sigma_{\mathrm{IN2}}^{-})$	√	×	×
	$(\sigma_{\mathrm{IN1}}^{-}, \sigma_{\mathrm{IN2}}^{+})$	√	×	
	$(\sigma_{\mathrm{IN1}}^{+}, \sigma_{\mathrm{IN2}}^{+})$	√	×	×
	$(\sigma_{\mathrm{IN1}}^{+}, \sigma_{\mathrm{IN2}}^{-})$	√	×	
Four-fold	$(\sigma_{\mathrm{IN1}}^{-}, \sigma_{\mathrm{IN2}}^{-})$	√	×	×
	$(\sigma_{\mathrm{IN1}}^{-}, \sigma_{\mathrm{IN2}}^{+})$	√	×	
	$(\sigma_{\mathrm{IN1}}^{+}, \sigma_{\mathrm{IN2}}^{+})$	√	×	×
	$(\sigma_{\mathrm{IN1}}^{+}, \sigma_{\mathrm{IN2}}^{-})$	√	×	
Three-fold	$(\sigma_{\mathrm{IN1}}^{-}, \sigma_{\mathrm{IN2}}^{-})$	√	√	√
	$(\sigma_{\mathrm{IN1}}^{-}, \sigma_{\mathrm{IN2}}^{+})$	×	×	
	$(\sigma_{\mathrm{IN1}}^{+}, \sigma_{\mathrm{IN2}}^{+})$	√	√	√
	$(\sigma_{\mathrm{IN1}}^{+}, \sigma_{\mathrm{IN2}}^{-})$	×	×	
Six-fold	$(\sigma_{\mathrm{IN1}}^{-}, \sigma_{\mathrm{IN2}}^{-})$	√	√	√
	$(\sigma_{\mathrm{IN1}}^{-}, \sigma_{\mathrm{IN2}}^{+})$	×	×	
	$(\sigma_{\mathrm{IN1}}^{+}, \sigma_{\mathrm{IN2}}^{+})$	√	√	√
	$(\sigma_{\mathrm{IN1}}^{+}, \sigma_{\mathrm{IN2}}^{-})$	×	×	
Five-fold	$(\sigma_{\mathrm{IN1}}^{-}, \sigma_{\mathrm{IN2}}^{-})$	√	×	×
	$(\sigma_{\mathrm{IN1}}^{-}, \sigma_{\mathrm{IN2}}^{+})$	×	×	
	$(\sigma_{\mathrm{IN1}}^{+}, \sigma_{\mathrm{IN2}}^{+})$	√	×	×
	$(\sigma_{\mathrm{IN1}}^{+}, \sigma_{\mathrm{IN2}}^{-})$	×	×	

The allowed nonlinear processes by m-fold rotational symmetry can be expressed as $\sigma_{\mathrm{OUT}}\hbar - \sigma_{\mathrm{IN}}\hbar = mN\hbar$ (N is an integer), where $\sigma_{\mathrm{OUT}}\hbar$ and $\sigma_{\mathrm{IN}}\hbar$ denote the spin angular momentum (corresponding to the chirality of light beam) of output and input beams, respectively; $mN\hbar$ denotes the angular momenta of the crystal lattice originating from the m-fold rotational symmetry. According to that, the allowed chiral FWM, SFG, and SHG processes in different materials are presented in Table S1. For SFG, it should be noted that they are only allowed in non-centrosymmetric materials. For SHG, it also requires centrosymmetry breaking, and the two photons with the same circular polarization are considered because we only consider the SHG with one incident beam. Note that the "√" and "×" in Table S1 denote the presence and absence of the output with the corresponding incidents.

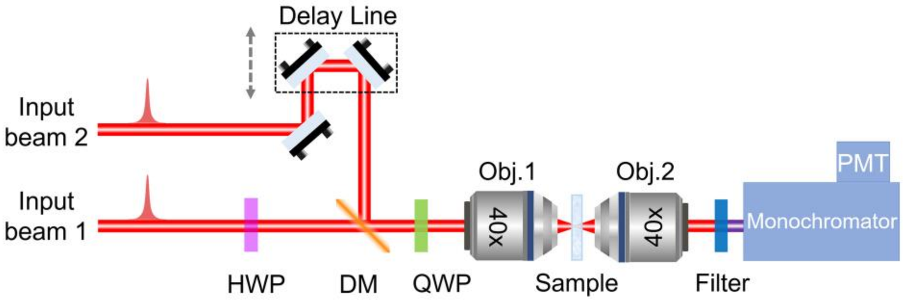

Figure S1 Schematic view of the experimental setup for characterizing the nonlinear effect of monolayer MoS_2. HWP: half-wave plate; DM: dichroic mirror; QWP: quarter-wave plate; obj.: objective lens; PMT: photomultiplier tube.

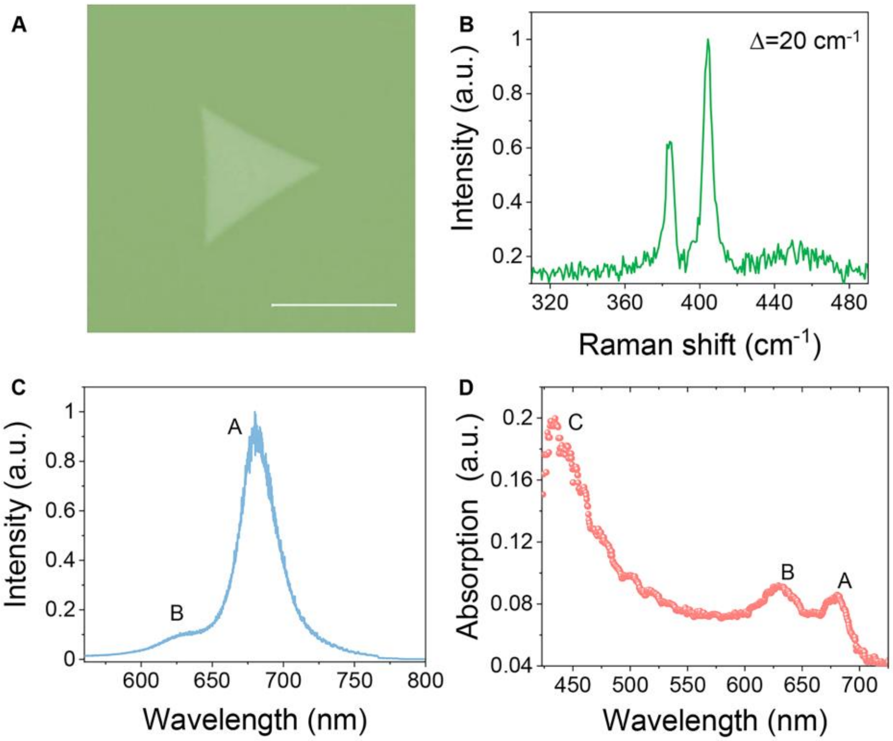

Figure S2 Characterization of monolayer MoS_2. (**A**) Optical image of monolayer MoS_2 flake on the quartz substrate. The scale bar is 10 μm. (**B**, **C**) Raman and PL spectra of monolayer MoS_2 with an excitation laser at the wavelength of 532 nm. (**D**) Optical absorption spectrum of monolayer MoS_2. Scale bar in (**A**) is 10 μm. The "A", "B" and "C" in (**C**, **D**) denote the A-, B- and C- exciton.

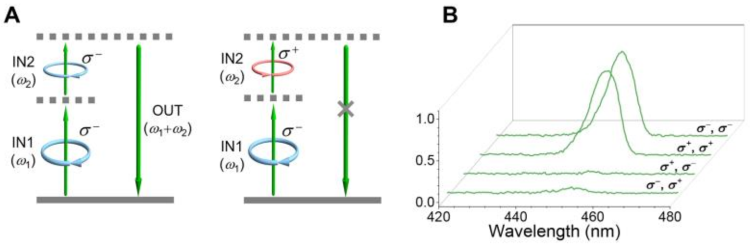

C

Input		Output
IN1	IN2	**XNOR**
0 (σ^-)	0 (σ^-)	1
1 (σ^+)	1 (σ^+)	1
1 (σ^+)	0 (σ^-)	0
0 (σ^-)	1 (σ^+)	0

Figure S3 Chirality XNOR logic gate based on sum frequency generation (SFG). (**A**) The optical chirality dependent selection rule in SFG. (**B**) The normalized SFG spectra measured in monolayer MoS_2 under the excitation of two input beams with different chirality combinations. (**C**) Truth table of the chirality XNOR logic gate. The measured conversion efficiency of SFG is ~1.7×10^{-6} % and second-order susceptibility $\left|\chi_{eff}^{(2)}\right|$ is ~2.1×10^{-10} m/V.

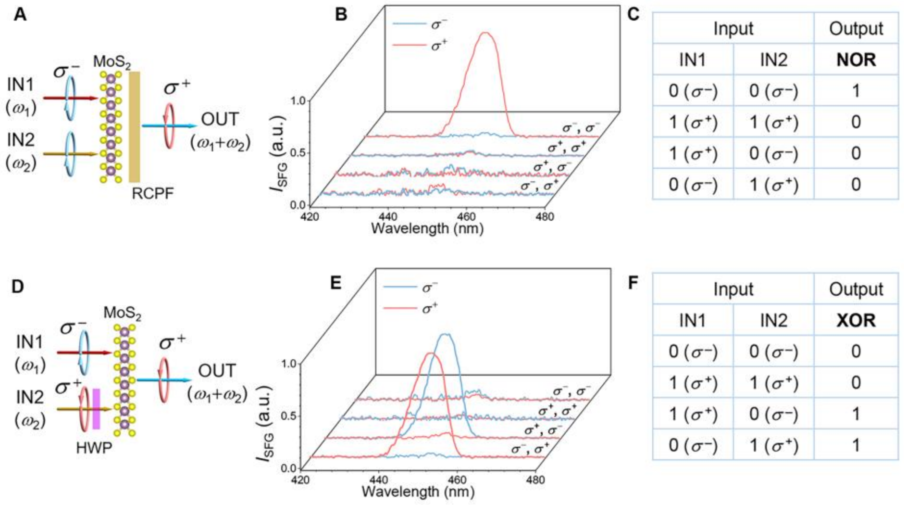

Figure S4 Diversity of chirality logic gate. The chirality of output spectra is analyzed by the combination of a quarter-wave plate and a polarizer. When the quarter-wave plate and polarizer combination allows the σ^- circularly polarized beam to pass through, it is called a left-handed circular polarization filter (LCPF). While the right-handed circular polarization filter (RCPF) refers to the quarter-wave plate and the polarizer combination allows the σ^+ circularly polarized beam pass through. (**A**) Schematic illustration, (**B**) Output SFG spectra with different chirality characteristics and (**C**) Truth table of NOR chirality logic gate. (**D**) Schematic illustration, (**E**) Output SFG spectra with chirality characteristics and (**F**) Truth table of XOR chirality logic gate. HWP: half-wave plate. The measured degree of circular polarization of the output signal beam is ~0.95.

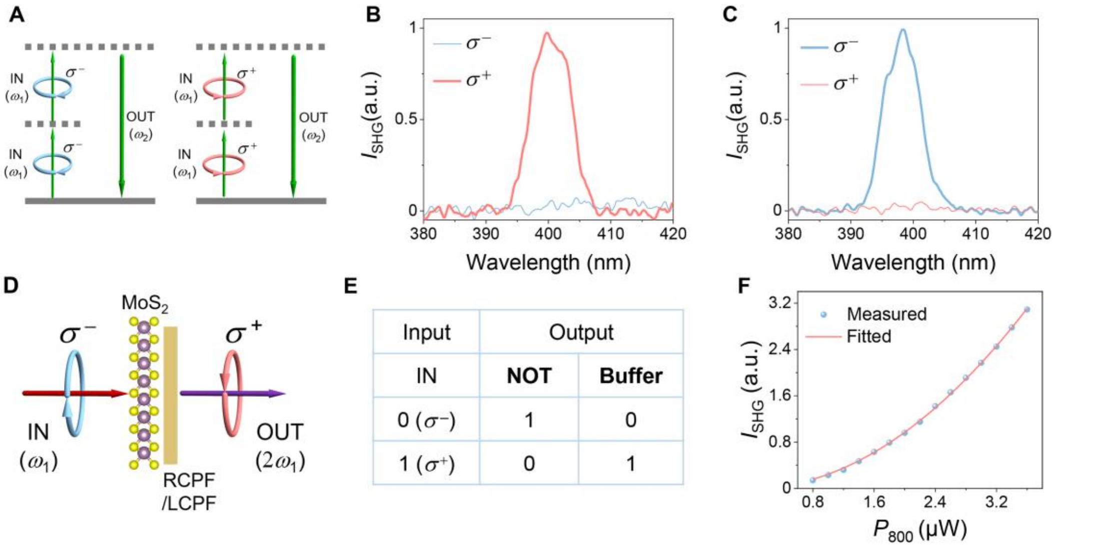

Figure S5 One-input chirality logic gates based on second harmonic generation (SHG). (**A**) The optical chirality dependent selection rule in SHG; (**B**, **C**) Output SHG spectrum with chirality characteristics when the input beam is σ^- and σ^+ circular polarization, respectively; (**D**) Schematic illustration of one-input chirality logic gates with SHG. (**E**) The truth table of NOT and Buffer gates. (**F**) Dependence of the peak intensity of SHG on the power of the input beam, where the fit (red curve) of the experiment data (blue spheres) is a power law I^s, s~1.98. The measured conversion efficiency of SHG is ~5.8×10^{-6} %.

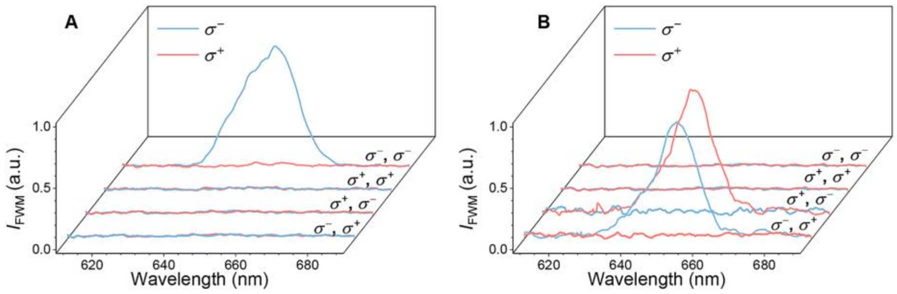

Figure S6 Output four-wave mixing (FWM) spectra with different chirality characteristics in monolayer MoS_2. (**A**) chirality resolved FWM spectra after inserting LCPF behind MoS_2. (**B**) chirality resolved FWM spectra in monolayer MoS_2 after inserting the HWP in the path of input beam 2. The measured degree of circular polarization of the output signal beam is ~0.93.

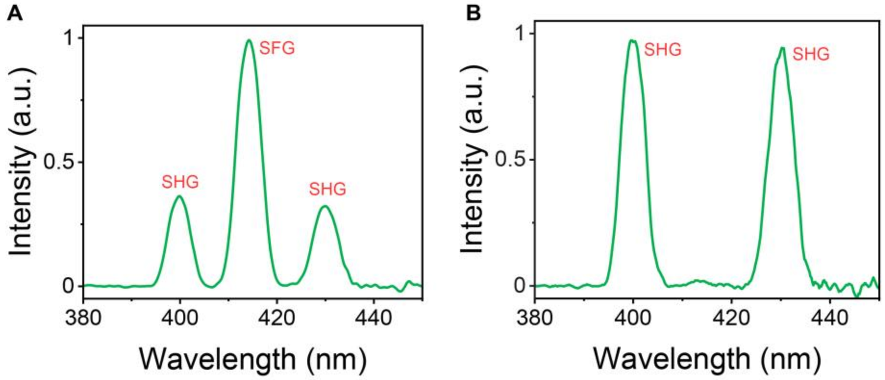

Figure S7 SHG and SFG spectra when the wavelengths of the two input beams are ~800 nm (~2 μW) and ~860 nm (~2 μW), respectively. (**A**) The two input beams are both σ^{+} circularly polarized. (**B**) The input beam 1 and input beam 2 are σ^{+} and σ^{-} circular polarization, respectively.

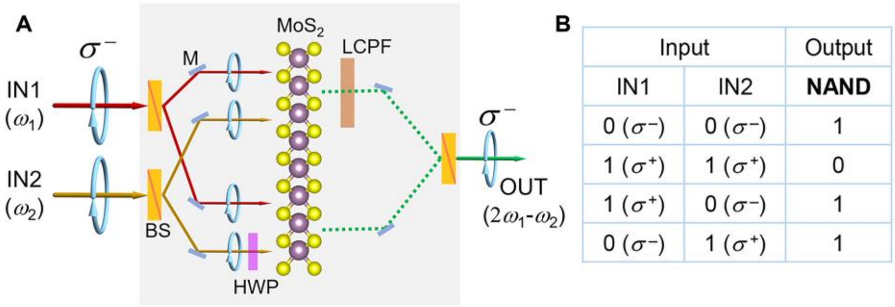

Figure S8 Construction of chirality NAND logic gate. (**A**) Schematic illustration of chirality NAND logic gate. M: high-reflection mirror; BS: beam splitter. (**B**) The truth table of NAND logic gate. Noted that the chirality OR logic gates can be constructed only by replacing the LCPF with an RCPF.

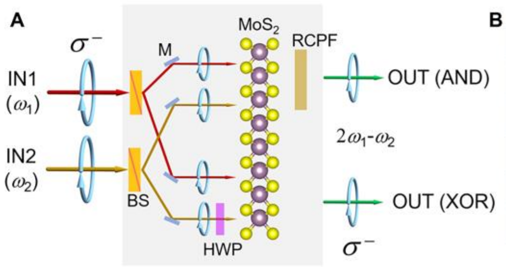

B

Input		Output	
IN1	IN2	**Sum**	**Carry**
0 (σ^-)	0 (σ^-)	0	0
1 (σ^+)	1 (σ^+)	0	1
1 (σ^+)	0 (σ^-)	1	0
0 (σ^-)	1 (σ^+)	1	0

Figure S9 Construction of a half adder. (**A**) Schematic illustration of the chirality half adder. Each input is split into two input beams by the BS. As a result, the two outputs can be viewed as the AND and XOR logic gates, which perform the addition operation. (**B**) The truth table of the half adder.

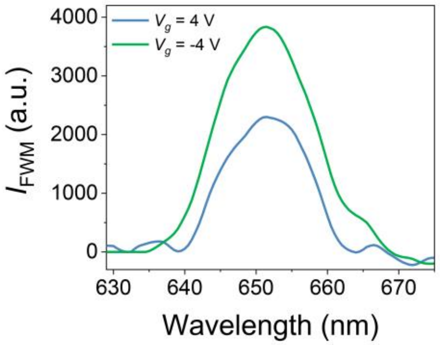

Figure S10 Measured FWM spectra. Spectra under different external voltages (V_g = -4 V and 4 V), where the input beams have both σ^+ circular polarizations at ~800 nm (~1.5 μW) and 1036 nm (~8.5 μW), respectively.

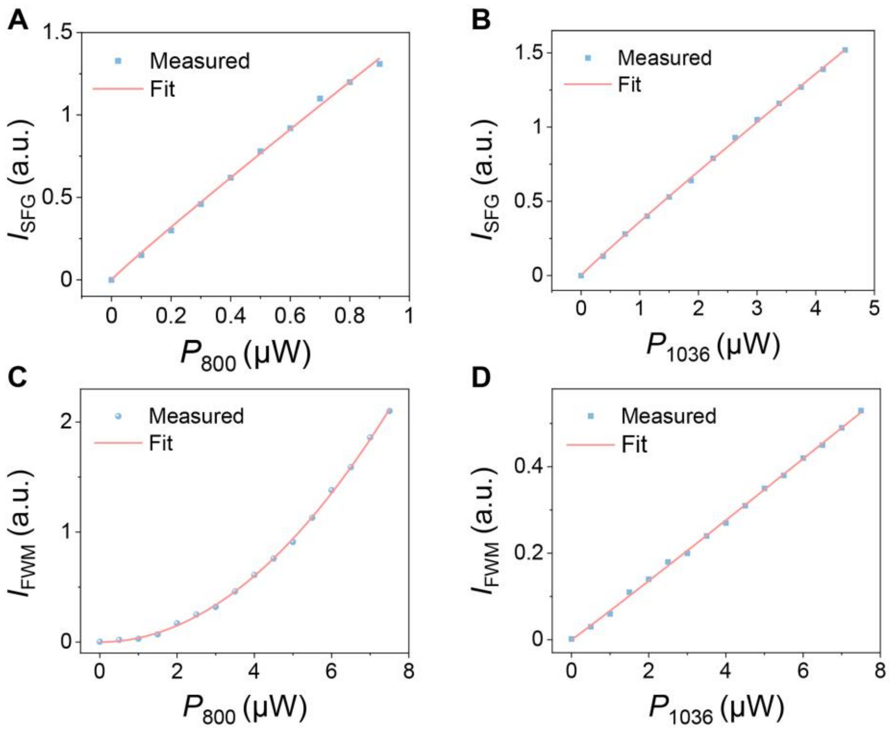

Figure S11 Power dependence of SFG and FWM. (**A**, **B**) Dependence of the peak intensity of SFG on the power of input beams, where the fit (red curves) of the experiment data (blue spheres) is a power law I^s, s~0.95 (P_{1036} is fixed at ~8 μW) in (**A**) and s~0.95 (P_{800} is fixed at ~2 μW) in (**B**). (**C**, **D**) Dependence of the peak intensity of FWM on the power of input beams, where the fit (red curves) of the experimental data (blue spheres) is a power law I^s, s~2.00 (P_{1036} is fixed at ~10 μW) in (**C**) and s~1.02 (P_{800} is fixed at ~5 μW) in (**D**).
